# PacBio single-molecule long-read sequencing shed new light on the complexity of the *Carex breviculmis* transcriptome

**DOI:** 10.1186/s12864-019-6163-6

**Published:** 2019-10-29

**Authors:** Ke Teng, Wenjun Teng, Haifeng Wen, Yuesen Yue, Weier Guo, Juying Wu, Xifeng Fan

**Affiliations:** 1 Beijing Research and Development Center for Grass and Environment, Beijing Academy of Agriculture and Forestry Sciences, Beijing, 100097 People’s Republic of China; 2 Department of Plant Biology, University of California, Davis, Davis, CA USA

**Keywords:** *Carex breviculmis*, SMRT sequencing, Alternative splicing events, LncRNA, Transcription factors

## Abstract

**Background:**

*Carex* L., a grass genus commonly known as sedges, is distributed worldwide and contributes constructively to turf management, forage production, and ecological conservation. The development of next-generation sequencing (NGS) technologies has considerably improved our understanding of transcriptome complexity of *Carex* L. and provided a valuable genetic reference. However, the current transcriptome is not satisfactory mainly because of the enormous difficulty in obtaining full-length transcripts.

**Results:**

In this study, we employed PacBio single-molecule long-read sequencing (SMRT) technology for whole-transcriptome profiling in *Carex breviculmis*. We generated 60,353 high-confidence non-redundant transcripts with an average length of 2302-bp. A total of 3588 alternative splicing events, and 1273 long non-coding RNAs were identified. Furthermore, 40,347 complete coding sequences were predicted, providing an informative reference transcriptome. In addition, the transcriptional regulation mechanism of *C. breviculmis* in response to shade stress was further explored by mapping the NGS data to the reference transcriptome constructed by SMRT sequencing.

**Conclusions:**

This study provided a full-length reference transcriptome of *C. breviculmis* using the SMRT sequencing method for the first time. The transcriptome atlas obtained will not only facilitate future functional genomics studies but also pave the way for further selective and genic engineering breeding projects for *C. breviculmis*.

## Background

Genus *Carex* L. consists of more than 2000 grassy species of the family Cyperaceae, commonly known as sedges, has a worldwide distribution in temperate and cold regions, and contribute constructively to turf management, forage production, and ecological preservation [1]. The wide application of transcriptome sequencing has promoted plant breeding and revealed gene regulation networks in plants [2]. However, few studies have focused on the transcriptome of *Carex* L., with previous studies being limited to physiological investigation and stress-resistance evaluation [3, 4]. Consequently, progress in the study of the transcriptome of the genus lags far behind. Thus, *Carex* L. breeding urgently needs a theoretical basis at the molecular level and further exploration of genetic resources. Although Li et al. (2018) firstly reported the salt-responsive mechanism of regulation in *Carex rigescens* utilizing next generation sequencing (NGS), the current description of that transcriptome remains unsatisfactory due to the inborn limitations of NGS technology in reads length.

The PacBio single-molecule long-read sequencing technology (SMRT sequencing) can obtain full-length splice isoforms directly, without assembly, thus providing a better opportunity to investigate genome-wide full-length cDNA molecules [5]. To date, SMRT sequencing has been successfully utilized in human-transcript cataloguing and quantifying [6, 7], as well as in various plant species, such as *Triticum aestivum* [8], *Oropetium thomaeum* [9], *Trifolium pretense* [10], *Medicago sativa* [11], *Fragaria vesca* [5], *Arabidopsis thaliana* [12] and *Phyllostachys edulis* [13]. These studies proved the power of SMRT sequencing in transcriptome analysis. With the help of NGS sequencing in error correction, SMRT sequencing may uncover full-length splicing isoforms with complete 3′ and 5′ ends more accurately, better identify differential alternative splicing (AS) events, and provide more accurate profiles of global polyadenylation sites (APA) [13, 14].

*Carex breviculmis* is a perennial grass with wide distribution in China that lives mainly under tree crowns, as it is highly shade tolerant. As afforestation in China accelerates, *C. breviculmis* is expected to be planted more widely. However, to date, the genetic resources of *C. breviculmis* have not been properly exploited, hampering the progress of *C. breviculmis* breeding efforts.

Aiming to provide a full-length reference transcriptome atlas for *C. breviculmis*, we generated high quality full-length non-chimeric reads (FLNC) in the present study by taking advantage of SMRT sequencing technology combined with NGS sequencing methods. In addition, AS events and long non-coding RNAs (lncRNAs) were predicted. Our results provided new insights into the possible mechanism underlying the transcriptional regulation of shade tolerance in *C. breviculmis*.

## Results

### General properties of PacBio sequencing of *C. breviculmis*

To provide a collection of gene transcripts, we combined the total RNA extracted from *C. breviculmis* grown under two different conditions (normal light and shade treatment) in equal amounts to obtain a full-length reference transcriptome using PacBio sequencing. Three cDNA libraries of different sizes (1–2 kb, 2–3 kb and 3–6 kb) were constructed and then sequenced using the PacBio RSII sequencing platform, thereby generating 11.52 Gb of SMRT sequencing raw data consisting of 751,460 raw polymerase reads. These reads resulted in 5,086,638 post-filter subreads (length > 50 bp and accuracy > 0.75) with an average of 1,017,327 subreads per cell (Table 1).

**Table 1:** SMRT sequencing statistics

Sample Name	cDNA Size	SMRT Cells	Polymerase Reads	Post-Filter Polymerase Reads	Post-Filter Total Number of Subread Bases	Post-Filter Number of Subread	Post-Filter Subreads N50	Post-Filter Mean Subread length
F01	1-2 K	2	300,584	212,898	4,534,764,579	2,686,464	1684	1688
F01	2-3 K	2	300,584	224,496	4,408,863,727	1,739,464	2631	2534
F01	3-6 K	1	150,292	119,219	2,578,445,894	660,710	4022	3902
F01	All	5	751,460	556,613	11,522,074,200	5,086,638	–	–

**Fig. 1:**
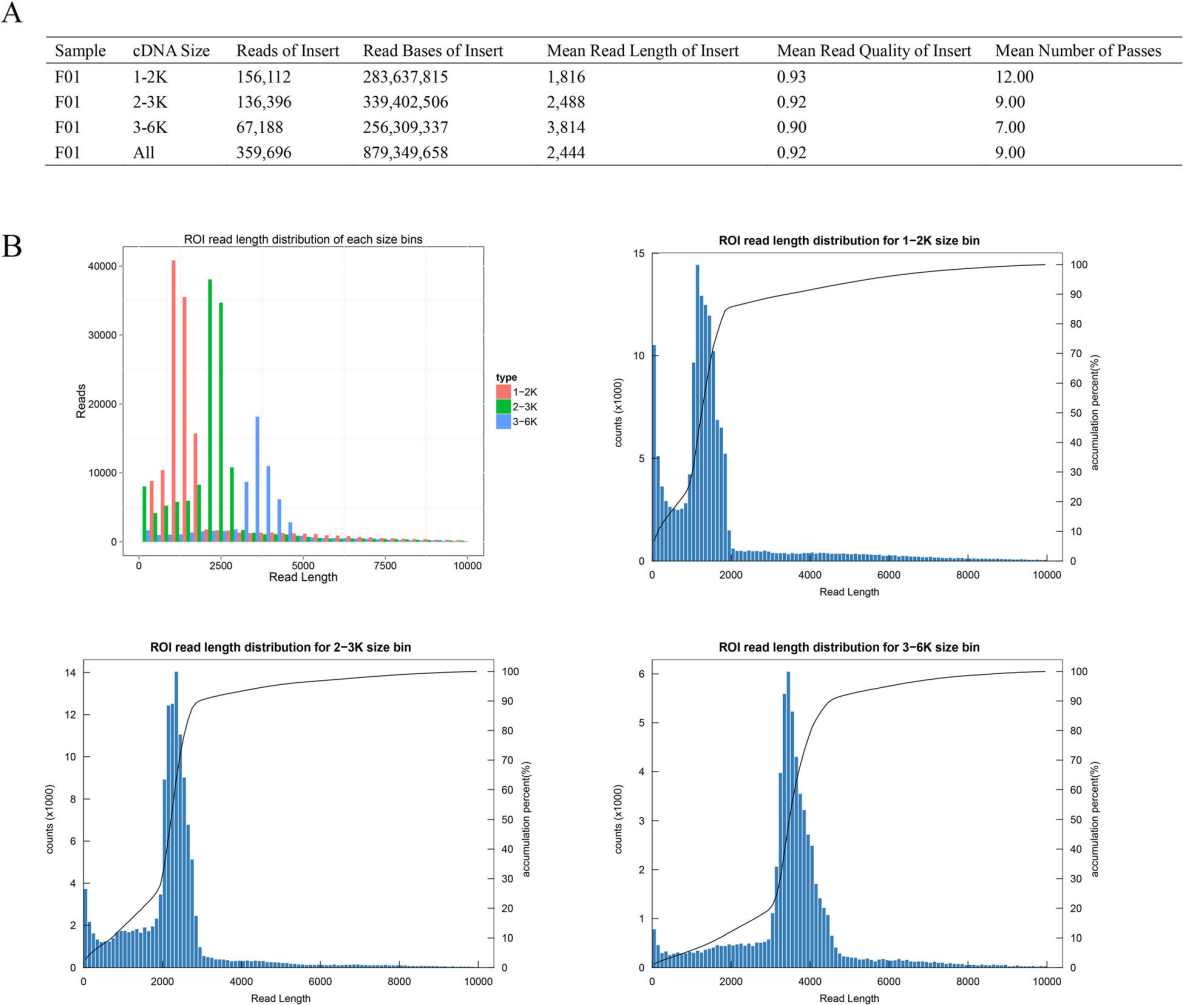
Statistics of Read of Insert (ROI). **a** Summary of ROI. **b** ROI read length distribution of each size bins

**Fig. 2:**
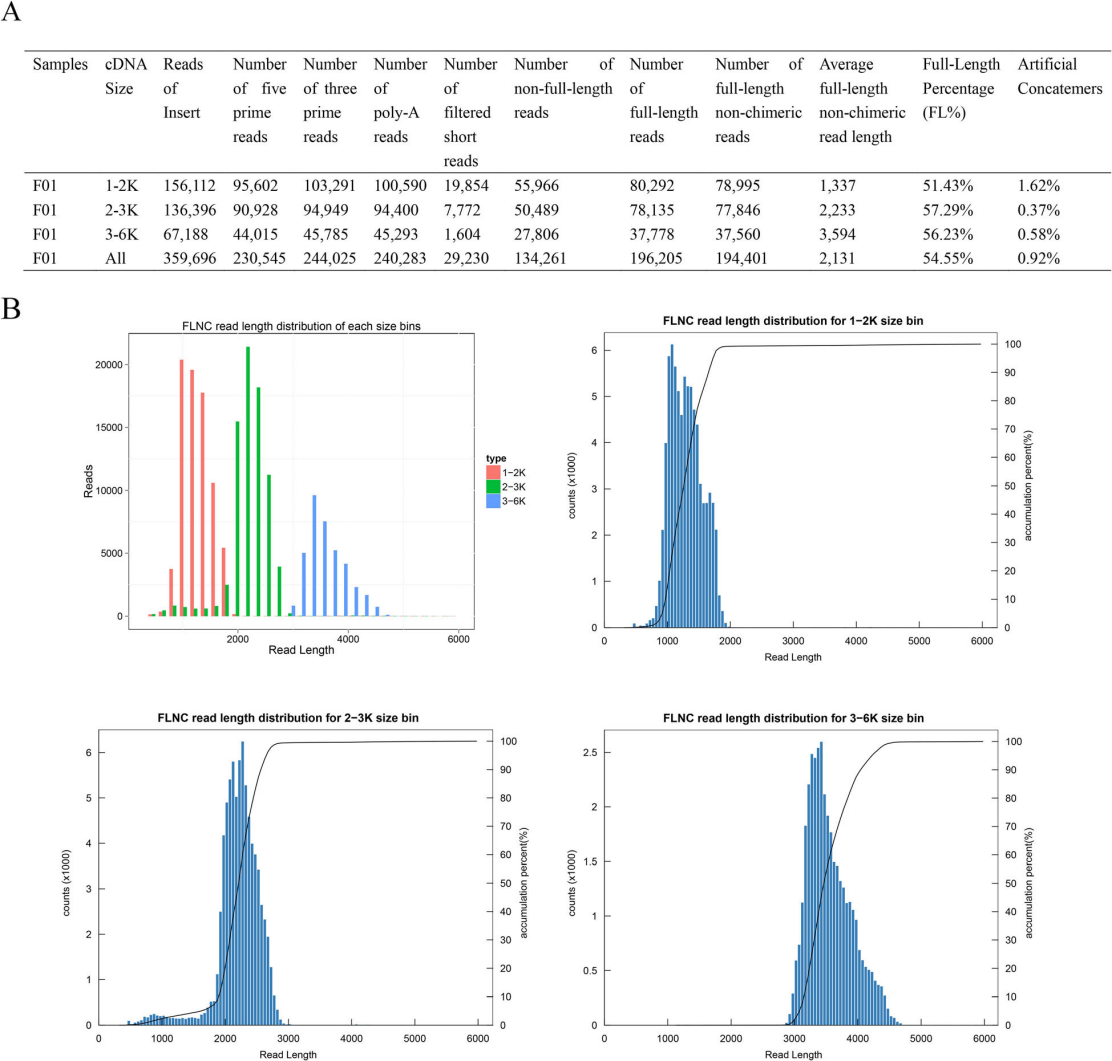
Statistics of full length sequences (FL). **a** Summary of FL. **b** FLNC reads length distribution of each size bins

Five single molecular real-time cells generated 156,112, 136,396 and 67,188 reads of insert (ROIs) from each of the three libraries, respectively (Fig. 1a). As expected, the ROIs mean length was consistent with each size-selected library (Fig. 1b). The mean number of passes in the three cDNA libraries was 12, 9 and 7, respectively. Among the 359,696 ROIs generated, more than 54.55% (194,401) were FLNC reads comprising the entire transcript region from the 5′ to the 3′ end based on the inclusion of barcoded primers and 3′ poly (A) tails (Fig. 2a). The FLNC read-length distribution of each size bin agreed with the size of its cDNA library (Fig. 2b). Short reads with a length < 300 bp (8.13%) and chimeric reads (0.92%) were discarded from subsequent analysis.

**Fig. 3:**
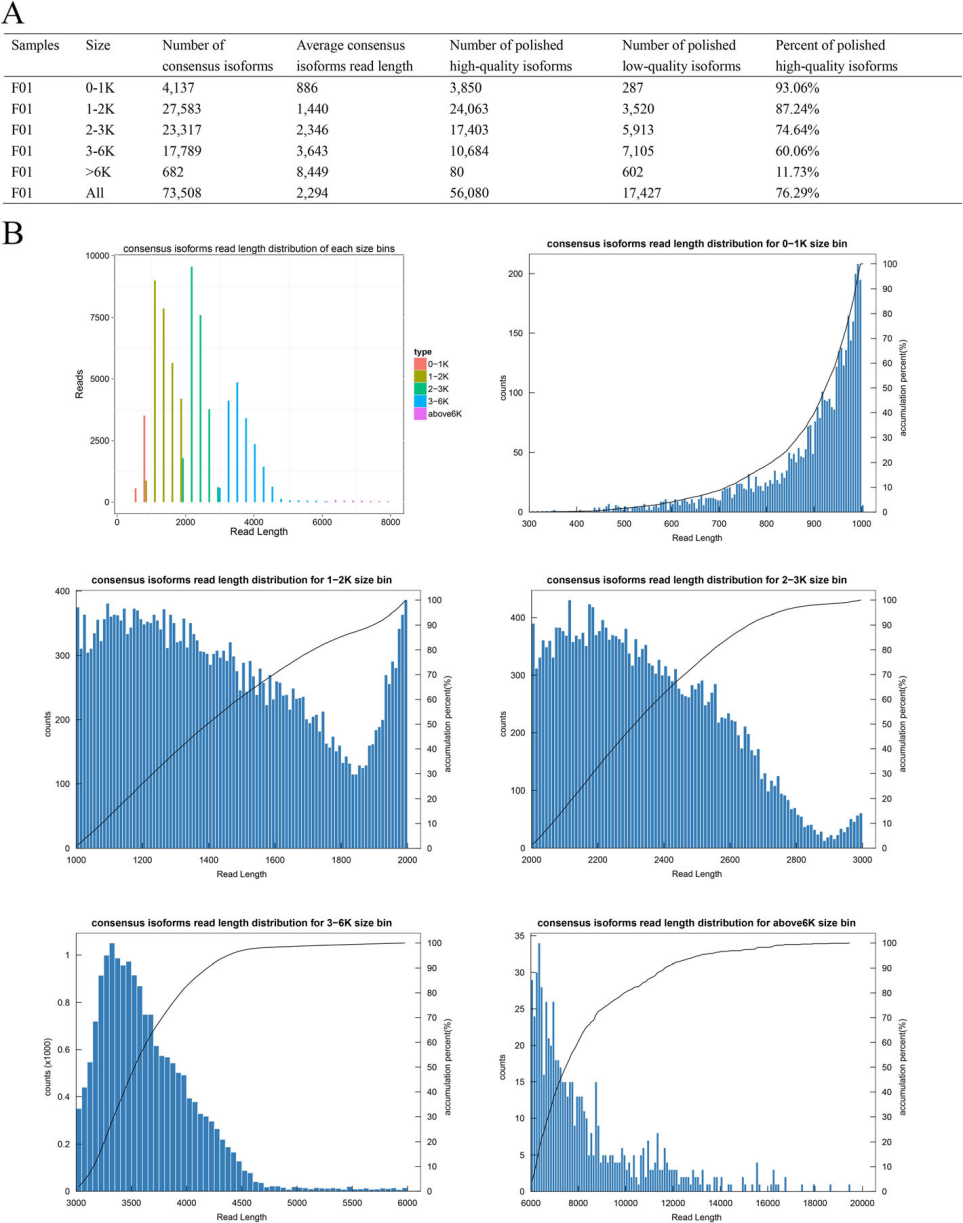
Statistics of consensus isoforms generated by ICE program. **a** Summary of consensus isoforms. **b** Consensus isoforms read length distribution of each size bins

The 73,508 consensus FLNC reads were first clustered using Iterative isoform-clustering program (ICE) program and then polished using the quiver program and non-full-length (NFL) reads. We obtained 56,080 high-quality isoforms (HQ) from 73,508 consensus isoforms (Fig. 3a). The read-length distribution of consensus isoforms in each size bin was in line with their sizes (Fig. 3b). To correct the relative high error rates of single-molecule long-reads compared with the Illumina platform, we generated 43.67 Gb of NGS raw sequencing data. Next, 146,112,446 paired-end reads (PE) were utilized to further polish the 17,427 low-quality isoforms (LQ) (Table 2). With the HQ transcripts and corrected LQ transcripts, we finally generated 60,353 high-quality non-redundant transcripts of *C. breviculmis* using the CD-HIT software. The average length of the 60,353 transcripts was 2302-bp, and the N50 value was 2547-bp. The most abundant transcripts were distributed in the length range > 3000 bp (25.5%), while transcripts in the 300–400 bp range accounted for the least percentage (0.02%). Particularly, the shortest transcript was 305-bp (F01.PB2138) while the longest was 24,616-bp (F01.PB60208).

**Table 2:** The results of NGS data mapped to SMRT transcriptome reference

Sample	Total Reads	Mapped Reads	Uniq mapped Reads	Multi mapped Reads
T01	23,149,717 (100%)	19,221,348 (83.03%)	2,549,937 (13.27%)	16,671,411 (86.73%)
T02	25,090,746 (100%)	20,692,371 (82.47%)	2,802,822 (13.55%)	17,889,549 (86.45%)
T03	32,835,048 (100%)	26,848,588 (81.77%)	3,583,241 (13.35%)	23,265,347 (86.65%)
T04	21,150,781 (100%)	16,731,511 (79.11%)	2,102,845 (12.57%)	14,628,666 (87.43%)
T05	22,232,685 (100%)	17,463,262 (78.55%)	2,184,655 (12.51%)	15,278,607 (87.49%)
T06	21,653,469 (100%)	17,062,209 (78.80%)	2,205,225 (12.92%)	14,856,984 (87.08%)

### Analysis of alternative splicing events

One of the most important advantages of SMRT sequencing is its ability to identify AS events by directly comparing isoforms of the same gene. Here, we performed a systematic analysis of AS in *C. breviculmis* based on high-quality full-length isoforms. The results showed that 5052 AS events were identified among the transcripts which had two or more alternative isoforms (Additional file 4: Table S1). Further analysis showed these AS events consisted of seven alternative splicing types, being retained intron (RI) the most abundant type with 2790 occurrences.

### Classification of long non-coding RNAs and their target genes

**Fig. 4:**
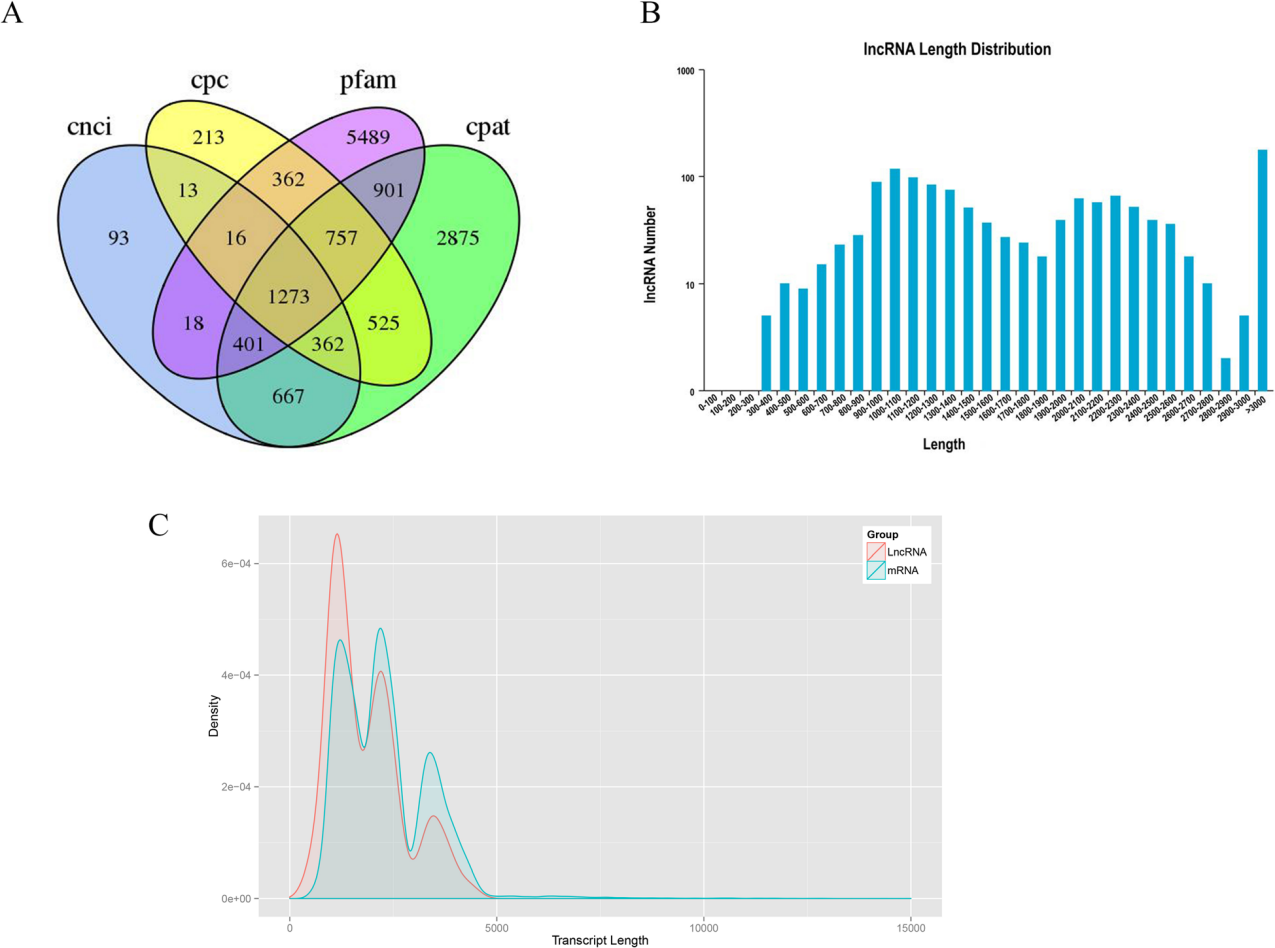
Prediction of lncRNAs. **a** Candidate lncRNAs predicted by CPC, CNCI, pfam and CPAT databases. **b** Length distribution of lncRNAs. **c** Comparison of lncRNA and mRNA length distribution

Based on the prediction of Coding Potential Calculator (CPC), Coding-Non-Coding Index (CNCI), Protein family (pfam) and Coding Potential Assessment Tool (CPAT), 13,965 transcripts were primarily found to be putative non-coding RNAs (Fig. 4a). Finally, 1273 candidates (with length greater than 200 bp and having more than two exons) which could be found in all the four prediction results, are believed to be lncRNAs (Additional file 5: Table S2). Length distribution analysis of lncRNAs revealed their lengths ranged from 0.317 kb (PB2821) to 7.93 kb (PB60053) with a mean length of 1.86 kb (Fig. 4b). The N50 of these identified lncRNAs was 2208 bp. Length distribution of protein coding mRNA showed that their lengths ranged from 0.305 kb (PB2138) to 24.62 kb (PB60208) with a mean length of 2.31 kb. Comparison results proved that mRNAs were significant longer than lncRNA in length (Fig. 4c). Moreover, 230 lncRNAs were predicted to have target mRNAs (Additional file 6: Table S3). Particularly, PB2554 had 98 target mRNAs, which was the largest number of target mRNAs attributed to any lncRNA.

### Prediction of coding sequences and functional annotation

**Fig. 5:**
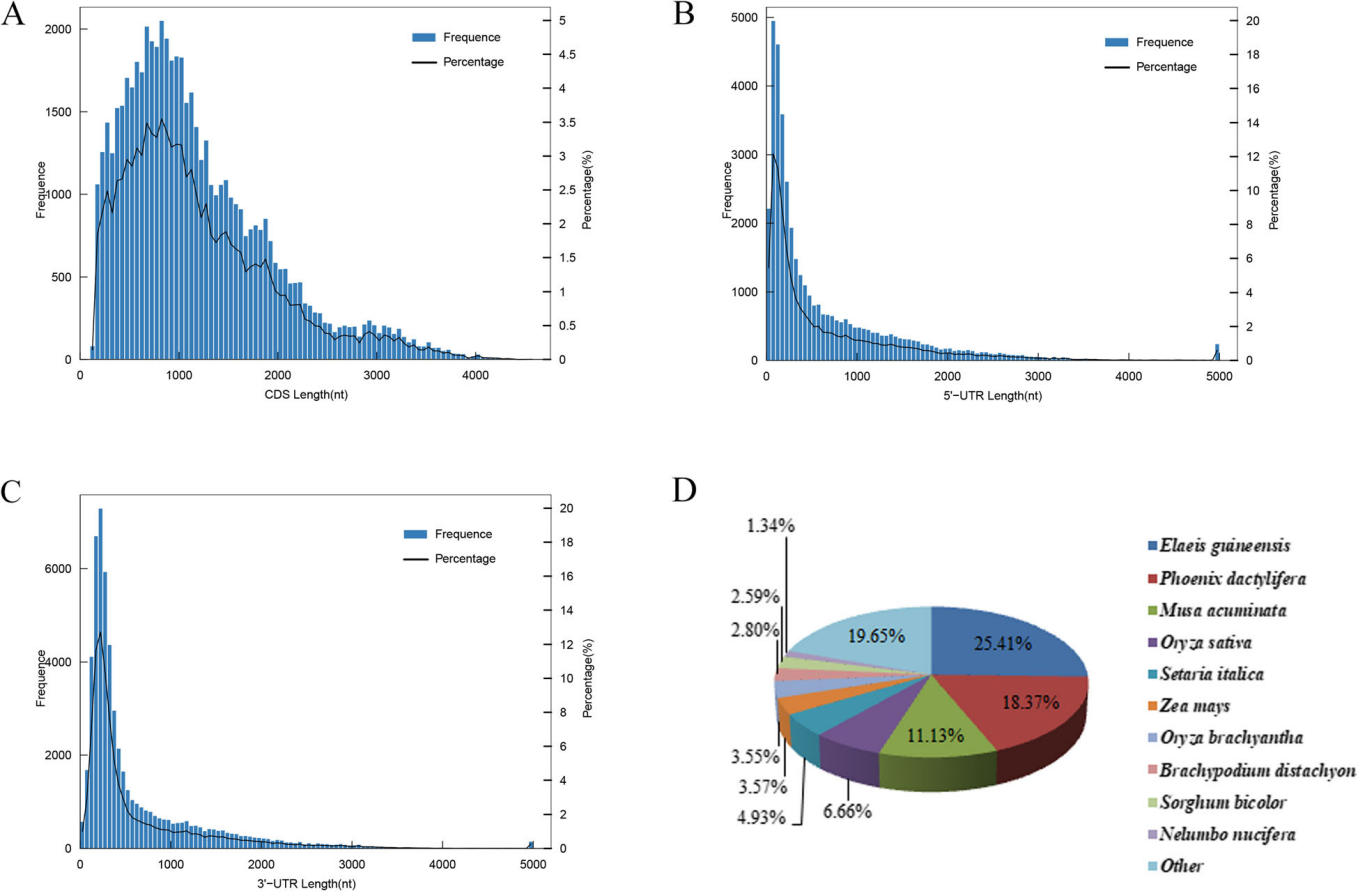
CDS-UTR structure analysis of SMRT sequences and NR annotation. **a** Length distribution of the complete transcripts. **b** Length distribution of the 5′-UTR. **c** Length distribution of the 3′-UTR. **d** NR protein alignments of *C. breviculmis* unigenes

The TransDecoder program was used to predict coding sequence (CDS) and untranslated regions (UTRs). These unique full-length transcripts involved 57,816 CDS with a mean length of 1189.23 nucleotides, including 40,347 transcripts with complete open reading frames (ORFs) (data not shown). Full-length transcripts consisting of 600–900 nucleotides were most abundant and corresponded to19.89% of the identified CDS (Fig. 5a). In addition, the results provided 418 3′ partial UTRs with a mean length of 974.96 bp and 16,963 5′ partial UTRs with a mean length of 1295.96 bp (Fig. 5b-c). To get insight into the reliability of the full-length transcripts of *C. breviculmis*, the CDS-containing transcripts generated by SMRT were used as queries against those of rice. The results showed that 68.57% (39,644 of 57,816) of the transcripts identified in *C. breviculmis* were homologous to those of rice, while the other 31.43% (18,172 of 57,816) were specific to *C. breviculmis*. The homologous transcripts and *C. breviculmis* specific transcripts are listed in Additional file 7: Table S4.

Using the basic local alignment search tool (BLAST) on several databases, 60,353 non-redundant transcripts were annotated for the reference transcriptome. In general, 42,604, 27,264, 39,038, 49,017, 43,321, 27,160, 57,429, and 58,130 transcripts were annotated in the GO, KEGG (Kyoto Encyclopedia of Genes and Genomes), KOG (euKaryotic Orthologous Groups), Pfam (a database of conserved protein families or domains), Swissprot (a manually annotated, non-redundant protein database), COG (Clusters of Orthologous Genes), eggNOG (evolutionary genealogy of genes: Non-supervised Orthologous Groups) and NR (NCBI non-redundant protein databases), respectively. Finally, based on the annotation results, 58,328 integrate annotated transcripts were generated, providing a comprehensive reference transcriptome for *C. breviculmis.* In addition, NR protein alignments results showed that 25.41% of the sequences could be aligned to *Elaeis guineensis*, followed by *Phoenix dactylifera* (18.37%) and *Musa acuminate* (11.13%) (Fig. 5d).

### Shade treatment caused significant changes in photosynthetic parameters in *C. breviculmis*

**Fig. 6:**
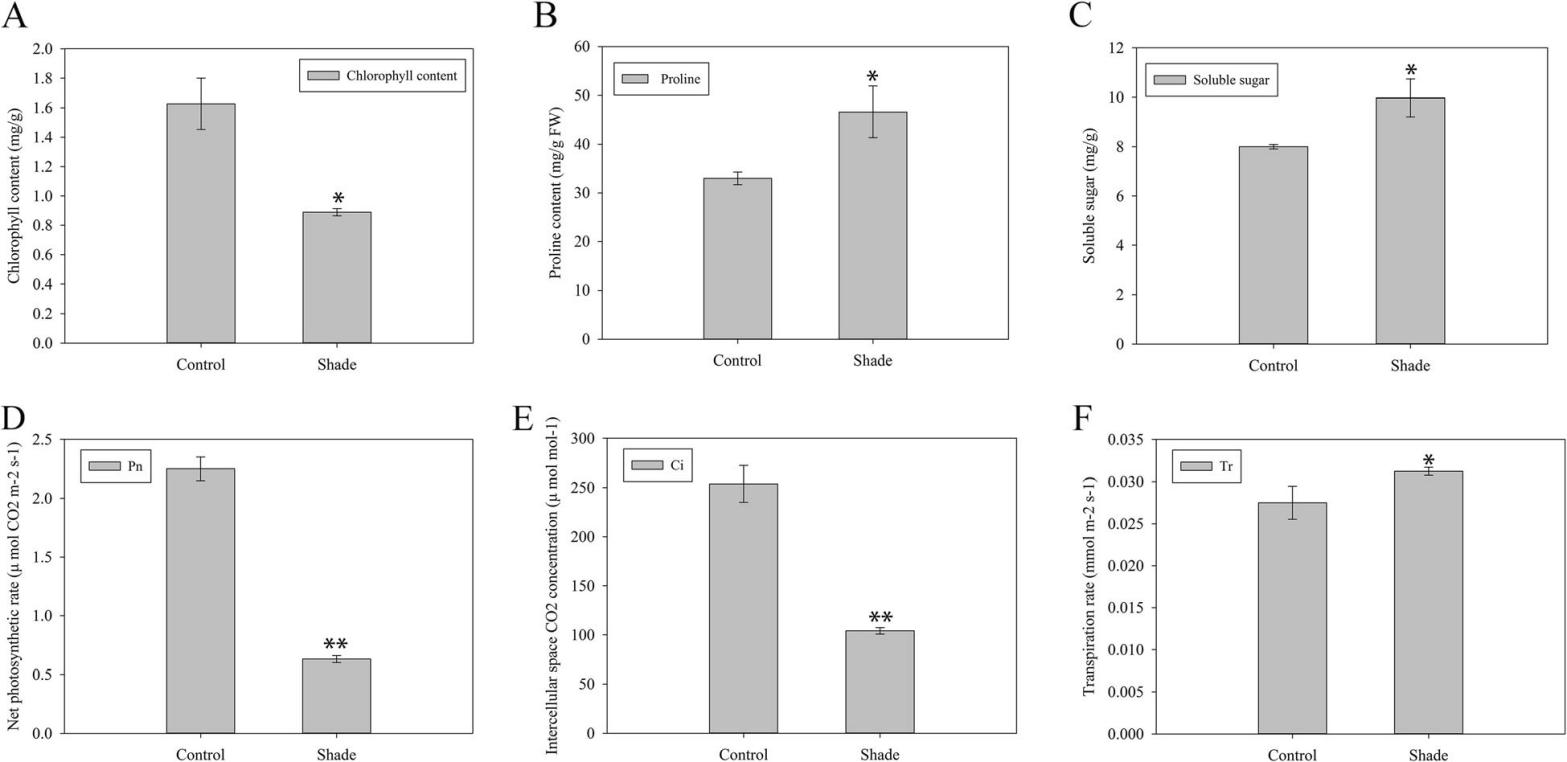
Physiological change of *C. breviculmis* in responses to shade treatment. **a** Chlorophyll content. **b** Proline content. **c** Soluble sugar content. **d** Net photosynthetic rate (Pn). **e** Intercellular CO_2_ concentration (Ci). **f** Transpiration rate (Tr). ∗ and ∗∗, respectively, represent significant differences from the control at values of *p* < 0.05 and *p* < 0.01 as determined by Student’s *t*-test

Shortages of light can cause physiological as well as structural changes in plants. We investigated several physiological traits associated with shade tolerance to determine the appropriate sampling time. The results showed that shade treatment reduced chlorophyll content but increased proline and soluble sugar contents (Fig. 6a-c). Photosynthetic parameters including net photosynthetic rate (Pn), intercellular space CO_2_ concentration (Ci), transpiration rate (Tr) and stomatal conductance (Cd) were examined to investigate the photosynthetic changes induced by shade treatment. Overall, shade stress reduced Pn and Ci, but increased Tr (Fig. 6d-f). However, no obvious change in Cd was observed (data not shown). The results above evidenced that a two-week shade treatment was sufficient to significantly alter the photosynthetic performance of *C. breviculmis*, indicating that this period was a suitable sampling time for sequencing analysis.

### Global gene expression analysis revealed transcriptional responses of *C. breviculmis* to shade stress

**Fig. 7:**
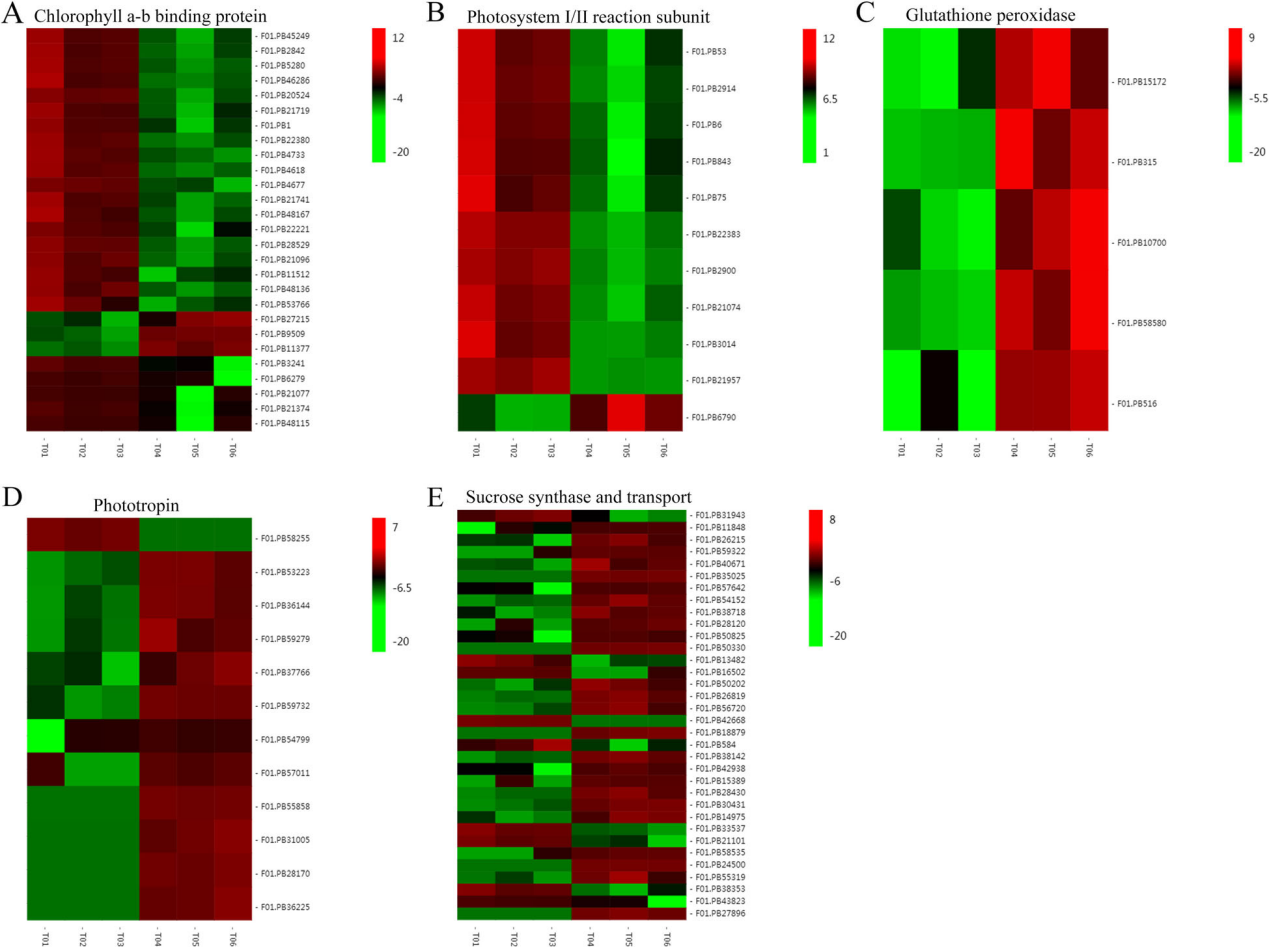
Heatmaps of the differentially expressed genes (DEGs) involved in shade responses in *C. breviculmis*. **a** Chlorophyll a-b binding proteins. **b** Photosystem I/II reaction subunit. **c** Glutathione peroxidase. **d** Phototropin. **e** Sucrose synthase and transport

Samples were validated for further analysis after examining the dependency of biological repetitions (Additional file 1: Figure S1A-B). As shown in Additional file 2: Figure S2A, 2926 of the 6514 differentially expressed genes (DEGs) identified were up-regulated while 3588 were down-regulated under shade conditions, compared to control. qRT-PCR experiments were carried out to examine the reliability of RNA-seq data using 10 randomly selected DEGs, and results obtained were in agreement with the digital expression results, thereby demonstrating the accuracy of our data analysis on global expression (Table 3, Additional file 8: Table S5). Subsequently, we then analyzed the genes presenting the highest differences in expression between both conditions. The top 10 up-regulated and down-regulated DEGs were listed in Table 4. We adopted GO annotation of the DEGs to better definite their functions. GO analysis showed that DEGs were classified into 53 groups based on their allocated gene ontology terms (Additional file 2: Figure S2B). Within the biological process category, ‘metabolic process’ was the most over-represented GO term. ‘Cell’ and ‘catalytic activity’ ranked the most over-represented among ‘cell component’ and ‘molecular function’ categorise, respectively. Furthermore, GO classification within biological process revealed that ‘response to absence of light’, ‘proline transport’, ‘chlorophyll biosynthetic process’ and ‘photosystem II assembly’ were significantly over-represented (Additional file 9: Table S6). Specifically, DEGs related to ‘Chlorophyll a-b binding’ and ‘Photosystem I/II reaction subunit’ exhibited a down-regulation tendency (Fig. 7a-b); while DEGs related to ‘Glutathione peroxidase’ and ‘Phototropin’ showed an up-regulation trend (Fig. 7c-d).

**Table 3:** The results of qRT-PCR using ten randomly selected genes

Gene annotation	ID	Tendency	Shade/Control (log2 FC)	
			Digital	qPCR
STAY-GREEN protein	F01.PB13572	Up	2.98	3.46
Chlorophyll a-b binding protein	F01.PB45249	Down	−2.44	−1.62
photosystem I reaction center subunit V	F01.PB53	Down	−1.72	−1.33
phospholipid hydroperoxide glutathione peroxidase 1	F01.PB15172	Up	2.14	1.76
root phototropism protein 2	F01.PB53223	Up	2.95	4.32
sucrose synthase 4	F01.PB11848	Up	3.84	2.41
WRKY transcription factor 70	F01.PB46640	Down	−4.38	−3.14
ethylene-responsive transcription factor ERF118	F01.PB53027	Up	1.11	1.61
scarecrow-like protein 21	F01.PB23994	Down	−2.15	−2.09
NAC domain-containing protein 100	F01.PB57159	No DEG	–	0.53

**Table 4:** The top 10 up-regulated and down-regulated genes in gene expression analysis

ID	log2FC	FDR	Regulated	Swissprot annotation
F01.PB17844	13.20	9.34E-73	up	Isocitrate lyase
F01.PB19517	11.96	3.66E-06	up	ATP-dependent zinc metalloprotease FTSH 1, chloroplastic
F01.PB19655	11.76	1.37E-04	up	Malate synthase
F01.PB9089	11.67	3.06E-91	up	Malate synthase
F01.PB55857	11.25	6.22E-07	up	Saccharopine dehydrogenase
F01.PB1057	10.97	6.88E-12	up	BTB/POZ and TAZ domain-containing protein 1
F01.PB57591	10.91	1.51E-3	up	Cyclin-dependent kinase F-4
F01.PB14534	10.59	3.21E-09	up	Hsp70-Hsp90 organizing protein 3
F01.PB15763	10.53	1.24E-10	up	ATP-dependent zinc metalloprotease FTSH 12, chloroplastic
F01.PB666	10.41	6.61E-79	up	Asparagine synthetase
F01.PB21933	−12.07	5.82E-94	down	3′-N-debenzoyl-2-deoxytaxol-N-benzoy ltransferase
F01.PB30266	−11.20	2.64E-41	down	Wall-associated receptor kinase 2
F01.PB58435	−10.55	2.03E-30	down	Receptor-like serine/threonine-protein kinase SD1–8
F01.PB54131	−9.90	2.73E-12	down	Protein translocase subunit SECA1, chloroplastic
F01.PB43948	−9.30	4.30E-26	down	Cinnamoyl-CoA reductase 1
F01.PB4894	−9.23	9.40E-53	down	High-affinity nitrate transporter 2.3
F01.PB1704	−9.21	1.39E-15	down	Bidirectional sugar transporter SWEET4
F01.PB3797	−9.16	7.70E-11	down	uncharacterized protein
F01.PB10550	−9.05	3.29E-23	down	Anthocyanidin 5,3-O-glucosyltransferase
F01.PB52691	−8.87	3.76E-33	down	Wall-associated receptor kinase 2

Then, all the DEGs were KEGG annotated to shed light on the pathways underlying the response to shade tolerance. In general, 50 KEGG pathways were classified (Additional file 2: Figure S2C). In detail, ‘Carbon metabolism’, ‘Biosynthesis of amino acids’ and ‘Starch and sucrose metabolism’ ranked as the top three pathways in terms of the number of annotated genes. Moreover, in order to reveal which pathways might be responsible for shade tolerance in *C. breviculmis*, we performed the statistical analysis of pathway enrichment. Statistically, ‘Alanine, aspartate and glutamate metabolism’, ‘Diterpenoid biosynthesis’, ‘Tropane, piperidine and pyridine alkaloid biosynthesis’ were the top three enriched pathways (Additional file 2: Figure S2D). Additionally, ‘Nitrogen metabolism’, ‘Starch and sucrose metabolism’ and ‘Photosynthesis - antenna proteins’ were also statistically enriched in the top 10 pathways and the DEGs responsible for ‘Sucrose synthase and transport’ were up-regulated, which was consistent with the pathway enrichment results (Fig. 7e).

### Transcription factor dynamics during shade responses of *C. breviculmis*

**Fig. 8:**
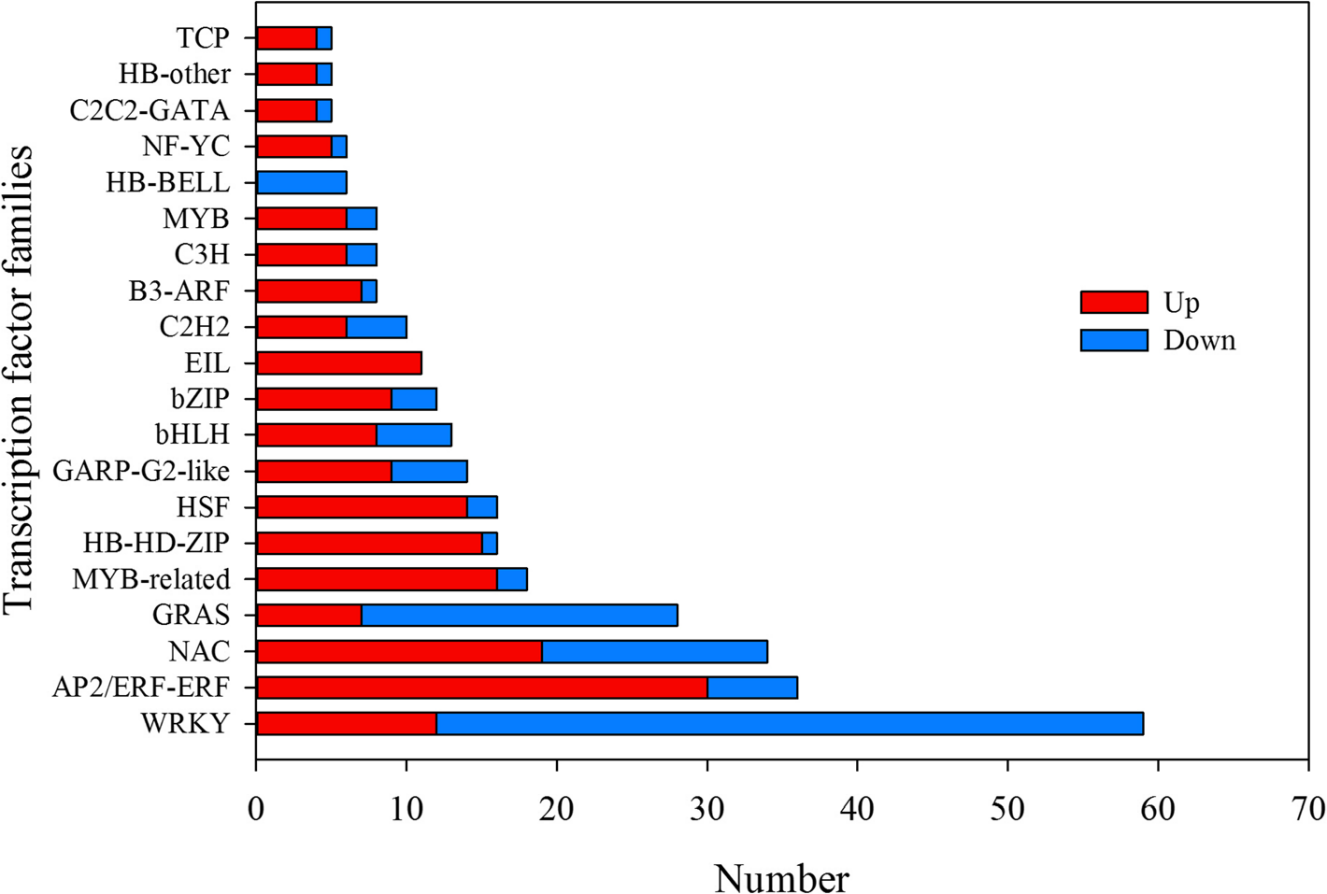
The differentially expressed transcription factors (TFs) involved in shade responses in *C. breviculmis*

Transcription factors (TFs) function as key players in plant responses to biotic or abiotic stresses conditions. Rationally, we focused on the expression patterns of TFs in responses to shade stress of *C. breviculmis.* Among 2953 TFs expressed in the sample sequenced in this study, 363 of them were monitored to be differentially expressed in responses to shade tolerance (Additional file 10: Table S7). Furthermore, TFs were classified into 40 families utilizing HMMER3.0 and Arabidopsis TF database TFBD 3.0 as reference (Fig. 8). Results revealed that under shade treatment, 227 TFs were up-regulated, while 136 were down-regulated. The number of down-regulated members in the WRKY TF family, which was the most abundant TF family identified in this study, was almost 4-fold larger than the number of up-regulated ones (Additional file 3: Figure S3A). More down-regulated TFs were also found in the GRAS and HB-BELL families (Additional file 3: Figure S3B-C). Nevertheless, up-regulated TFs were more abundant than down-regulated TFs in other TF families, such as AP2-ERF, NAC, and MYB-related TFs families (Additional file 3: Figure S3D-F).

### Assemble of the unaligned Illumina short reads

We assembled the ~ 20% Illumina short reads which were not aligned with the full-length transcriptome build in this study using reference free method. The results contained 56,920 unigenes, among which 32,031 unigenes could be annotated. BLAST analysis was carried out using the unigenes against the full-length transcriptome constructed in this study. Finally, 34,663 unigenes were proved to have not been detected by SMRT sequencing. The assemble results of the Illumina short reads was provided in Additional file 11: Table S8.

## Discussion

*Carex*, one of the largest genus in Cyperaceae, contains more than 2000 species and plays important roles in turfgrass and forage production as well as in ecological conservation [1]. However, our current knowledge on *Carex* species transcriptomes is mainly based on gene expression analysis of *Carex rigescens* using NGS technologies [3]. Moreover, the *Carex.* transcriptome had not been fully explored due to lack of full-length cDNA. In this study, we adopted the SMRT sequencing strategy with assistance of NGS sequencing to provide a comprehensive transcriptomic landscape of *C. breviculmis* by generating 11.52 Gb of raw data from 5 SMRT cells and 43.67 Gb of NGS raw data. After clustering, correction, and redundancy elimination, we finally obtained 60,353 high-quality non-redundant transcripts were obtained from *C. breviculmis*. This information provides the first reported SMRT sequencing atlas of the full-length transcriptome of *C. breviculmis*.

In diverse biological responses, AS serves as an effective mechanism to increase the complexity and flexibility of the entire transcriptome and proteome [5]. Because of the advantage in long-read sequencing, SMRT sequencing enabled accurate identification of the complexity of AS at genome-wide level [14]. In this study, 5.95% (3588/60,353) of transcripts were identified to be alternatively spliced, suggesting that AS occurs in *C. breviculmis*, although with lower frequency than that found in strawberry [5], bamboo [13], and Arabidopsis [12]. This lower frequency might be partially due to the lack of a reference genome, suggesting that there may be other isoforms to explore. Recent estimates suggest that 13 to 18% of the intron-containing genes are regulated by AS and non-sense-mediated decay in Arabidopsis roots [15]. Further accurate classification of the AS types based on the availability of a reference genome will further uncover the types of AS events and shed light on post-transcriptional regulation in *C. breviculmis*.

lncRNAs, a recently identified class of non-coding RNAs function as essential regulators in a wide range of biological processes [16, 17]. By using NGS sequencing, most studies aiming at identifying lncRNAs inevitably generated lncRNA transcripts lacking poly(A) tails, thereby affecting data accuracy [18]. In the present study, 1273 lncRNAs were predicted based on SMRT sequencing data, providing candidates for future functional characterization. The average length of lncRNA was about three times longer than those identified in red clover [10]. Consistent with previous study, the lncRNAs were found to be shorter than the protein coding mRNAs in length [19].

The LncTar has advantages in accuracy and running speed in predicting lncRNA targets, providing a valuable and efficient tool for large-scale prediction of the targets of candidate lncRNAs [20]. In this study, 230 of 1273 lncRNAs were predicted to have target mRNAs by LncTar tool, providing an opportunity for comprehensively understanding the lncRNA functions in *C. breviculmis*. However, we did not validate the authenticity of the prediction results, so further experimental confirmation of the targets is still needed in the future.

Using the rice transcriptome as reference, 68.57% of the CDS-containing transcripts of *C. breviculmis* generated by SMRT were considered homologous to that of rice. This revealed the reliability of the full-length sequences obtained by SMRT and encouraged the functional annotation of the full-length transcripts which provided a reference transcriptome for *C. breviculmis* including 58,328 integrate annotated transcripts. NR alignment results showed that 25.41% of sequences could be aligned to *Elaeis guineensis*, indicating that *C. breviculmis* was most closely related to *E. guineensis* in terms of protein alignment based on the current NR database*. E. guineensis* belongs to *Palmae* while *C. breviculmis* belongs to *Cyperaceae.* Considering plant taxonomy, *E. guineensis* and *C. breviculmis* seem distantly related. The un-expected protein alignment results may, at least in part, be due to the lack of *Cyperaceae* related species data in the current NR database, reflecting the urgent need to improve the genetic database for this genus.

Light irradiance levels are known to affect the photosynthetic capacity and chlorophyll content in plants [21]. Stay-green proteins (SGRs) play key roles in chlorophyll degradation and photosystem stability [22]. In this study, reduction in chlorophyll content and photosynthetic efficiency were consistent with the expression level of the stay-green gene (F01.PB13572) in *C. breviculmis*. Chlorophyll a-b binding proteins are probably the most abundant membrane protein complexes and play important roles in light-harvesting activities in higher plants. Photosystem I/II reaction subunit mediates light-driven electron transfer, protecting plants from phototoxic damage [23]. The suppressed transcription of chlorophyll a-b binding protein and photosystem I/II reaction subunit, together with reduced chlorophyll content and reduced photosynthetic efficiency, reflected a subsequently damage caused by shade treatment.

*C. breviculmis* is widely used as an important cover plant in China because of its outstanding level of shade tolerance [24]. However, the molecular mechanisms underlying this tolerance remains largely unknown. Recent bioinformatics studies demonstrated that transcriptional signature can be captured by analyzing a subset of the most representative DEGs [25]. Two ATP-dependent zinc metalloprotease genes (*FTSH1* and *FTSH12*) and two malate synthase isoforms ranked the top 10 up-regulated genes. The FTSHs are correlated with in vivo photosystem II function [26], and malate synthase gene expression is activated in senescent cotyledons [27]. Wall-associated receptor kinase 2 is required for the activation of numerous genes in protoplasts [28]. Two wall-associated receptor kinase 2 genes were found among the representative down-regulated genes, reflecting their possible roles in shade responsiveness. These results provide candidates for future exploration.

In this study, global expression analysis revealed that biological processes related to ‘proline transport’, ‘chlorophyll biosynthesis’, and ‘photosystem II assembly’ were shade responsive activities in *C. breviculmis*. Glutathione peroxidase (GPX) was reported to help photosynthetic organs reduce oxidative damage in transgenic tobacco seedlings under stressful conditions [29]. Phototropins, a kind of serine/threonine protein kinases activated by blue-light radiation, play an essential role in the perception of light gradients within plant tissues [30]. Thus, the induced transcription of GPX and phototropins might contribute to shade adaption in *C. breviculmis*, at least partly, at the transcriptional level*.* Transcription factors such as WRKY, AP2-ERF, NAC and GRAS have been extensively reported as key players in plant responses to biotic and abiotic stresses [31–35]. Comparatively, and to our best knowledge, limited information related to shade tolerance has been provided. These TFs showed different expression patterns in *C. breviculmis* leaves, indicating their various roles in the response of this specie to shade stress.

In addition, pathways related to ‘Nitrogen metabolism’, ‘Starch and sucrose metabolism’, and ‘Photosynthesis-antenna proteins’ seem to actively participate in *C. breviculmis* shade tolerance. Plants have evolved a complex sucrose transport reaction to avoid shade stress [36]. Sucrose phosphate synthase and sucrose transferase are key enzymes of sucrose metabolism [37]. The over-represented DEGs related to sucrose synthase and transport suggest that sucrose metabolism might contribute to *C. breviculmis* shade tolerance. To the best of our knowledge, this is the first transcriptome-wide investigation of *C. breviculmis* in response to shade stress using reference transcriptome data generated by SMRT sequencing.

Although SMRT sequencing has clear advantages over Illumina sequencing in investigating full length transcripts, it has distinct limitations in throughput [38]. In addition, the limitation of lower throughput is most obvious when performing large-scale DEG experiments. The ~ 20% Illumina short reads which were not aligned to the reference full-length transcriptome may result from the sequencing depth of the PacBio RSII platform. So further long-read RNA-seq studies with improved throughput are still needed in the future.

## Conclusion

Collectively, we have generated 60,353 high-quality non-redundant full-length transcripts, 3588 AS events, 40,347 complete CDS and 1273 lncRNAs of *C. breviculmis*. The analysis of NGS data elucidated on the regulatory transcription mechanism underlying this plant response to shade. This study provides, for the first time, a full-length transcriptome of *C. breviculmis* using the SMRT sequencing method. The transcriptome atlas generated here will facilitate future studies on functional genomics and provided basis for further selective and genetic engineering breeding of *C. breviculmis*.

## Methods

### Plant material and growth conditions

*C. breviculmis* cultivar ‘Four seasons’ (China accession No. S-SV-CL-006-2010) was identified and cultivated at the Beijing Research and Development Center for Grass and Environment, and it has undergone inbreeding for at least five generations. It was collected from the National Experiment Station for Precision Agriculture (Beijing, China) for sequencing due to its wide application area in Beijing. The plants were planted in 20 cm diameter, 20 cm deep plastic pots filled with nutrition medium containing peat, vermiculite and pearlite (1:1:1 in volume). Plants were cultivated in a growth chamber (RXZ-380D-LED; Ningbo Jiangnan Instrument Factory, Ningbo, China) at 28/24 °C(day/night), 60% relative humidity, 14-h photoperiod, and average photosynthetic active radiation (PAR) of 400 μmol m^− 2^ s^− 1^. Plants were weekly fertilized with half-strength Hoagland’s solution [39]. For shade treatment, four independent plants were subjected to shade treatment at 100 μmol m^− 2^ s^− 1^ m^− 2^ s^− 1^ for two weeks. Whole plants were respectively collected for RNA extraction on each sampling day.

### Library preparation and SMRT sequencing

Total RNA was extracted using the Plant RNA Kit (OMEGA, Georgia, USA, No. R6827–01). Equal amounts of RNA collected from ‘Four season’ plants at each sampling day (0, 14th day) were pooled together. The quantity and integrity of RNA samples were assessed in the NanoDrop ND-1000 spectrophotometer (NanoDrop Technologies, Delaware, USA) and in the 2100 Bioanalyzer (Agilent Technologies, California, USA), respectively. Qualified RNA samples were then used for constructing cDNA libraries. The SMARTer PCR cDNA Synthesis Kit (TaKaRa, Dalian, China) was utilized to synthesize full-length cDNA and cDNA fraction and length selection (1–2 kb, 2–3 kb, and > 3 kb) were performed using the BluePippin^Tm^ Size Selection System (Sage Science, Massachusetts, USA). These three SMRT libraries were generated at Biomarker Technology Co. (Biomarker, Beijing, China) using the Pacific Biosciences DNA Template Prep Kit 2.0. (Pacific Biosciences, California, USA) according to the standard protocol. SMRT sequencing was then performed on the Pacific Bioscience RS II (Pacific Biosciences, California, USA) platform at Biomarker Technology Co. (Biomarker, Beijing, China) using the protocol provided by the manufacturer.

### Illumina cDNA library construction and second-generation sequencing

Total RNA was extracted from three independent biological replicates, each comprising one independent plant subject to either. Next, the six prepared RNA samples prepared (three from control and three from shade treatment) were evaluated as described above. The strand-specific cDNA libraries were constructed using a NEBNext® Ultra™ RNA Library Prep Kit (NEB, Massachusetts, USA) following the manufacturer’s protocol. Qualified libraries were sent to Biomarker Technology Co. (Biomarker, Beijing, China) for second-generation sequencing on the Illumina HiSeq4000 platform (San Diego, CA, USA). To verify the digital expression level of RNA-seq data, a qRT-PCR was carried out to assess the expression levels of ten representative genes. Relative expression level was figured out using the comparative ΔΔ Ct method [40].

### Quality filtering, error correction and elimination of redundancy among SMRT long reads

ROIs were generated from SMRT subreads using the standard protocols in the SMRT analysis software suit (http://www.pacificbiosciences.com). FLNC and NFL reads were classified using RS-IsoSeq (v2.3) by identifying poly (A) signal and 5′ and 3′ adaptors. To identify all possible reads, a relaxed standard with a minimum full pass of 0 and an accuracy of 75% was adopted in the filtering panel. The FLNC reads generated from the same isoform were clustered into one consensus isoform using ICE. Subsequently, the consensus isoforms were further polished by the Quiver program with the assistance of NFL reads, resulting in HQ (accuracy> 99%) and LQ. The raw Illumina reads were filtered to remove adaptor sequences, ambiguous reads with ‘N’ bases, and low-quality reads. Filtered Illumina data were then used to polish the LQ reads using the proovread 213.841 software [41]. The redundant isoforms (identity < 0.9, coverage < 0.85) were eliminated using the CD-HIT program [42] without considering the 5′ difference. Finally, a high-quality transcript dataset without redundant transcripts of *C. breviculmis* was constructed.

### Analysis of alternative splicing events

In this study, we adopted IsoSeq AS de novo script to identify AS events based on available non-redundant transcripts. Briefly, FLNC transcripts were clustered using the Cogent software (v1.0) to generate a UniTransModel file. Subsequently, error-corrected non-redundant transcripts were mapped to UniTransModels using GMAP [43] (version 2017-11-15, parameters: -f2-allow-close-indels 0 -min-trimmed-coverage = 0.85 -min-identify = 0.90 -cross-species) to build the General Feature Format (GFF) file used to detect AS events in SUPPA using the default settings.

### Prediction of long non-coding RNAs and their target genes

Four computational approaches including CPC (version 1.0, parameters: default), CNCI (version 2.0, parameters: default), CPAT (version 1.2, parameters: default) and Pfam (version 1.5, parameters: default) databases were combined to sort non-protein coding RNA candidates from putative protein coding RNAs in the transcripts. Putative protein-coding RNAs were filtered out using a minimum length and exon number thresholds. As so, transcripts longer than 200 bp with more than two exons were selected as lncRNAs candidates and further screened using CPC/CNCI/CPAT/Pfam, as these tools can distinguish protein-coding from non-protein coding genes. Only the transcripts identified in the four databases were regarded as lncRNAs. To investigate the target genes of lncRNAs, the LncTar tool (version 1.0, parameters: ndG value = − 0.1) was utilized following two different strategies: the first was based on the localization of lncRNA and mRNA, whereas the second was based on the interactivity results of lncRNA and mRNA caused by base pairing.

### Functional annotation of transcripts and prediction of putative coding sequences

TransDecoder v2.0.1 (https://transdecoder.github.io/) was utilized to define the putative CDS of the 60,353 non-redundant transcripts. The predicted CDS were then annotated and confirmed by BLAST (E-value ≤1 e^− 5^). Those transcripts containing complete ORFs as well as 5′ and 3′ UTR were designated as full-length genes. The CDS-containing transcripts were used as queries against those of rice (reference genome MSU_v7.0) in Orthomcl software [44] (version 2.0.3, parameters: PercentMatchCutoff = 50%, EvalueExponentCutoff = 1e-5, mc1 = 1.5). Functional annotations were conducted using BLAST (version 2.2.26) against the NR, Swissprot, COG, KOG, Pfam, GO, eggNOG, and KEGG databases. For each transcript in each database, the functional information of the best matched sequence was assigned to the query transcript.

### Physiological measurements

Chlorophyll content was measured using ethanol based on the protocol of Teng et al. (2016). Proline content was examined using the 5-sulfosalicylic acid method, as described in a previous report [40]. Soluble sugar content was measured by the anthrone sulphuric acid method [45]. Photochemical efficiency, including Pn, Ci, Cd, and Tr was estimated using the 6400XT system (Li-Cor, Lincoln, USA) according to the instructions provided by the manufacturer.

### Quantification of gene expression levels and analysis of differential gene expression

The Illumina clean reads were mapped to the constructed *C. breviculmis* reference transcriptome using Bowtie2 program [46]. The unmapped Illumina short reads were then assembled using reference free strategy. Next, the abundance of expressed reads presents as fragments per kilobase of transcript per million mapped reads (FPKM) was calculated using RSEM [47]. After examining the correlation of biological replicates based on Pearson’s correlation coefficient, differential expression analysis was carried out on DESeq program [48]. The parameters adopted in this study were Fold Change ≥2 and FDR *<* 0.01. The identified DEGs were mapped to each term of the GO database (www.geneontology.org) and calculated using GOseq-R package [49]. The *p*-value for enrichment was corrected via Kolmogorov-Smirnov test and the corrected *p*-value of ≤0.05 was believed to be significantly enriched. KOBAS [50] was used to test the statistical enrichment of DEGs in KEGG pathways. Using the Arabidopsis transcription factors in Plant TFDB 3.0 [51] as the reference database, *C. breviculmis* TFs were predicted and distinguished using HMMER 3.0 [52].

## Supplementary information

**Additional file 1: Figure S1.** NGS sequencing data evaluation and biological repetitions dependency examination. A. Statistics of FPKM results for each sample. B. biological repetitions dependency examination.

**Additional file 2: Figure S2.** Analysis of differential expressed genes (DEGs) for NGS. A. Volcano plot of DEGs. B. GO classification. C. KEGG annotation. D. KEGG enrichment.

**Additional file 3: Figure S3.** Heatmap of representative transcription factors.

**Additional file 4: Table S1.** Summary of the candidate alternative splice (AS) events.

**Additional file 5: Table S2.** Summary of the lncRNAs.

**Additional file 6: Table S3.** Prediction results of the target mRNAs for lncRNAs.

**Additional file 7: Table S4.** The identified homologous transcripts between *C. breviculmis* and rice.

**Additional file 8: Table S5.** Sequences of the primers used for qRT-PCR verification.

**Additional file 9: Table S6.** GO classification of the biological processes.

**Additional file 10: Table S7.** Summary of the transcription factors predicted in this study.

**Additional file 11: Table S8.** Illumina short read assemble results.

## Data Availability

The PacBio SMRT reads and the Illumina NGS reads generated in this study have been submitted to the BioProject database of National Center for Biotechnology Information (accession number PRJNA488303 and PRJNA488506).

## Abbreviations

APA: Polyadenylation sites
AS: Alternative splicing events
CDS: Putative coding sequences
DEGs: Differentially expressed genes
FLNC: Full-length non-chimeric reads
GO: Gene ontology
HQ: High quality isoforms
ICE: Iterative isoform-clustering program
KEGG: Kyoto Encyclopedia of Genes and Genomes
lncRNA: long non-coding RNA
LQ: Low quality isoforms
NFL: Non-full-length
NGS: Next generation sequencing
ROI: Reads of insert
SMRT sequencing: The PacBio single-molecule long-read sequencing technology
TFs: Transcription factors
