# Supplementary figures and images for: PacBio single-molecule long-read sequencing shed new light on the complexity of the Carex breviculmis transcriptome
Source: BMC Genomics. 2019 Oct 29;20:789. doi: 10.1186/s12864-019-6163-6 (PMC6821003; doi:10.1186/s12864-019-6163-6)

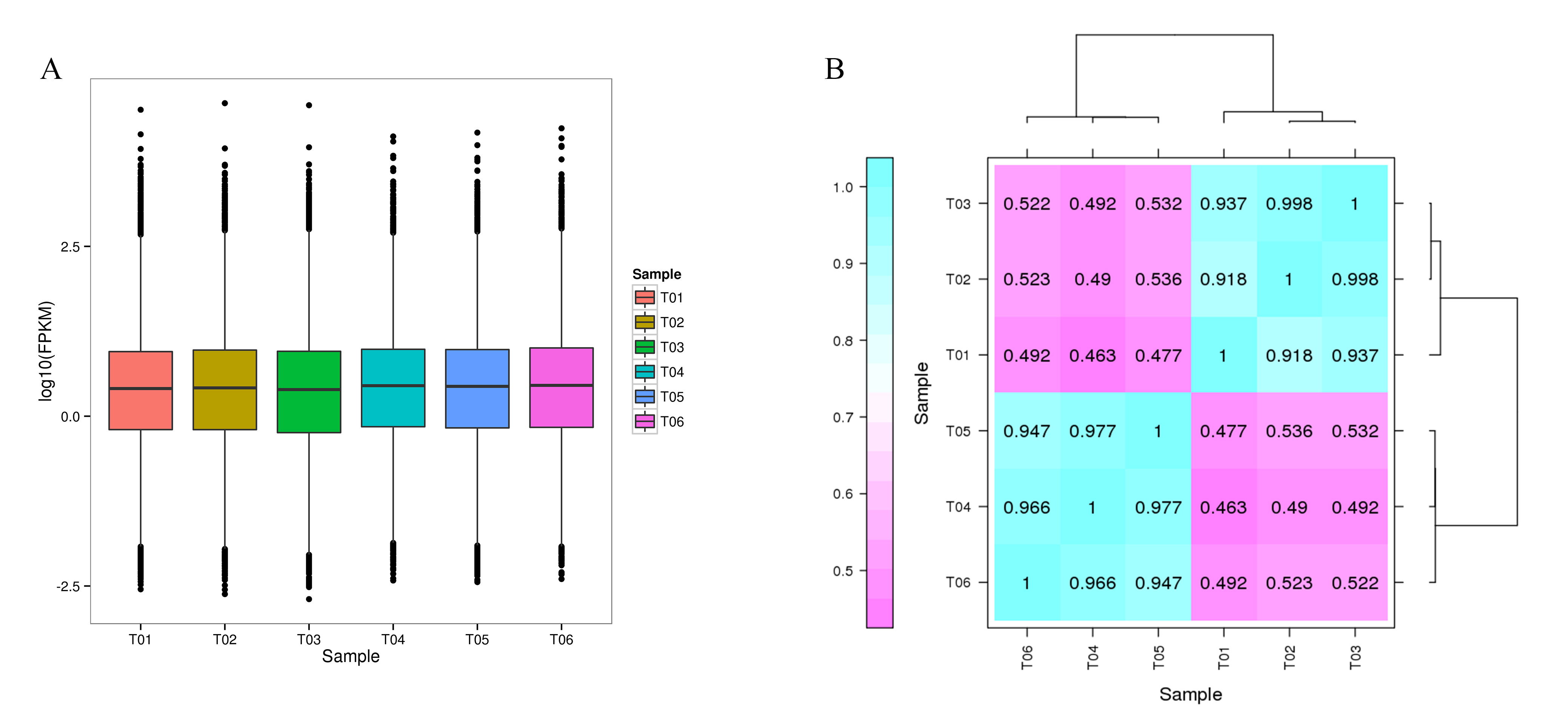

Supplement: Supplementary file 1 — Additional file 1: Figure S1. NGS sequencing data evaluation and biological repetitions dependency examination. A. Statistics of FPKM results for each sample. B. biological repetitions dependency examination. [file 12864_2019_6163_MOESM1_ESM.jpg]

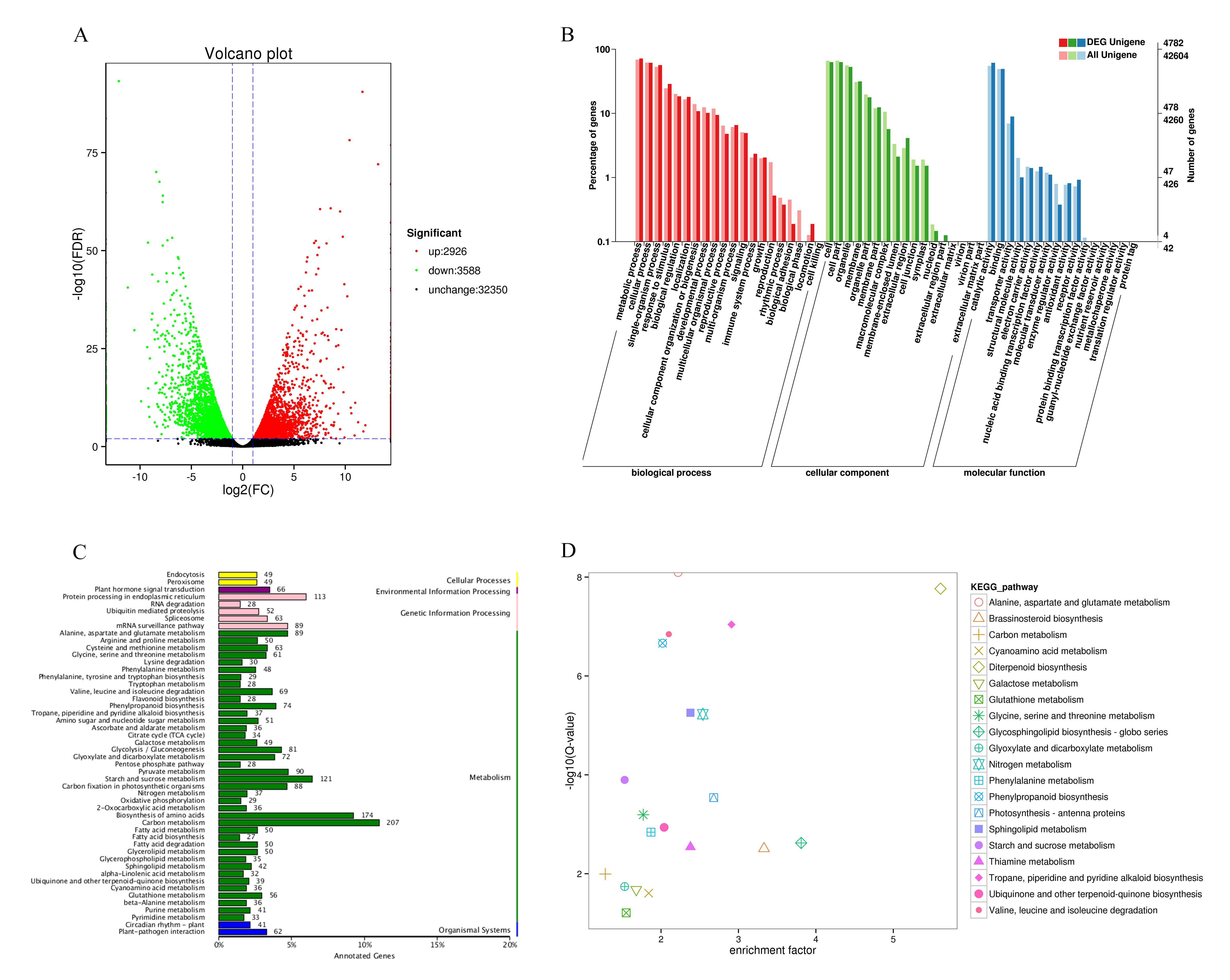

Supplement: Supplementary file 2 — Additional file 2: Figure S2. Analysis of differential expressed genes (DEGs) for NGS. A. Volcano plot of DEGs. B. GO classification. C. KEGG annotation. D. KEGG enrichment. [file 12864_2019_6163_MOESM2_ESM.jpg]

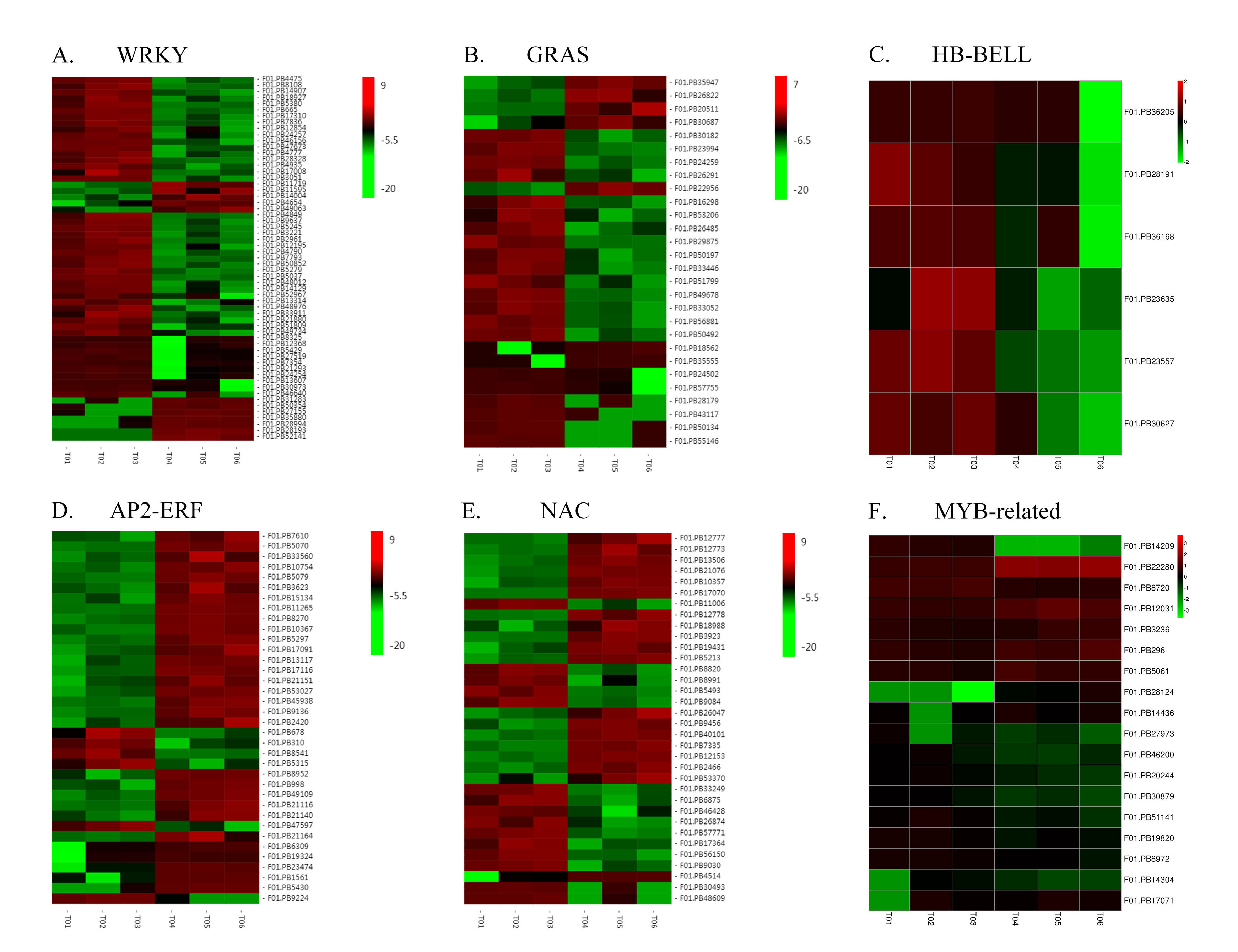

Supplement: Supplementary file 3 — Additional file 3: Figure S3. Heatmap of representative transcription factors. [file 12864_2019_6163_MOESM3_ESM.jpg]
