# Supplementary material for: PacBio single-molecule long-read sequencing shed new light on the complexity of the Carex breviculmis transcriptome
Source: BMC Genomics. 2019 Oct 29;20:789. doi: 10.1186/s12864-019-6163-6 (PMC6821003; doi:10.1186/s12864-019-6163-6)
Supplement: Supplementary file 8 — Additional file 8: Table S5. Sequences of the primers used for qRT-PCR verification. [file 12864_2019_6163_MOESM8_ESM.doc]

Table S5 Sequences of primers for qRT-PCR detection.

| Primer name | Primer sequence (5'-3') |
| --- | --- |
| qCbUBQ-F/R | CACCTTGTCCTGCGTCTCCG |
|  | GCGACCATCCTCCAACTGCT |
| qCbSGR-F/R | GGCAATGGTATGGGTTTACTTC |
|  | CTAACTTGTCGGGTTGTGGATC |
| qChla-b-F/R | CCCCGAAGCATTCAACAAAT |
|  | GCAGCTACAGCGACGACAAG |
| qCbPhotoI-F/R | CCAGCCAAGTTGCACTCATT |
|  | TTTCAGAAGACCGACATACTCC |
| qCbGPX-F/R | GACTTGTCGGGTCGTCTGTC |
|  | TTGCCCTTGAATTTGTTGAG |
| qCbPhototrop-F/R | CGAGAAGGGAAGCGGGTCTA |
|  | CTTTGCCACAGCCACATCGA |
| qCbSucrose-F/R | TGCCGAGGATGCAATAAGTGAG |
|  | TGCGAGCAAAGAAGCAACAAGA |
| qCbWRKY-F/R | CAAAGAAACATTGCCAAGAACA |
|  | TCCAAACCCAAGAAATTACTGC |
| qCbERF-F/R | GCATGTCAGGCCCACCAAGT |
|  | AGAAGCCGCAGCAGAGTCGT |
| qCbNAC-F/R | CTGGCAGAGTTATCTCGCTTCA |
|  | GGACAGGCATTGATCGCATC |
| qCbGRAS-F/R | CCACCGTGACCGCATCTTAC  CTCCACCCTGGTCCTGTCCT |
